# Newcastle disease virus degrades HIF-1α through proteasomal pathways independent of VHL and p53

**DOI:** 10.1099/jgv.0.000623

**Published:** 2016-12-15

**Authors:** Noraini Abd-Aziz, Eric J. Stanbridge, Norazizah Shafee

**Affiliations:** ^1^​ Department of Microbiology, Faculty of Biotechnology and Biomolecular Sciences, Universiti Putra Malaysia, UPM Serdang 43400, Malaysia; ^2^​ Department of Microbiology and Molecular Genetics, School of Medicine, University of California, Irvine, CA 92697, USA; ^3^​ Institute of Biosciences, Universiti Putra Malaysia, UPM Serdang 43400, Malaysia

**Keywords:** Newcastle disease virus, Hypoxia-inducible factor, von Hippel–Lindau, p53

## Abstract

Newcastle disease virus (NDV) is a candidate agent for oncolytic virotherapy. Despite its potential, the exact mechanism of its oncolysis is still not known. Recently, we reported that NDV exhibited an increased oncolytic activity in hypoxic cancer cells. These types of cells negatively affect therapeutic outcome by overexpressing pro-survival genes under the control of the hypoxia-inducible factor (HIF). HIF-1 is a heterodimeric transcriptional factor consisting of a regulated α (HIF-1α) and a constitutive β subunit (HIF-1β). To investigate the effects of NDV infection on HIF-1α in cancer cells, the osteosarcoma (Saos-2), breast carcinoma (MCF-7), colon carcinoma (HCT116) and fibrosarcoma (HT1080) cell lines were used in the present study. Data obtained showed that a velogenic NDV infection diminished hypoxia-induced HIF-1α accumulation, leading to a decreased activation of its downstream target gene, *carbonic anhydrase 9*. This NDV-induced downregulation of HIF-1α occurred post-translationally and was partially abrogated by proteasomal inhibition. The process appeared to be independent of the tumour suppressor protein p53. These data revealed a correlation between NDV infection and HIF-1α downregulation, which highlights NDV as a promising agent to eliminate hypoxic cancer cells.

## Introduction

Newcastle disease virus (NDV) is an avian paramyxovirus with an inherent oncolytic activity against mammalian cancers (Chia *et al.*, 2012, 2014; Jamal *et al.*, 2012). Despite being widely studied in the last few decades, the exact mechanism of its oncolytic ability is still unclear. Recently, we have shown that NDV was able to induce apoptosis in renal carcinoma cells by regulating the cellular antiviral response pathway (Ch’ng *et al.*, 2013). We observed that the oncolytic activity was significantly increased in hypoxic cancer cells. Hypoxic cancer cells upregulate pro-survival genes under the control of the hypoxia-inducible factor (HIF) (Lee *et al.*, 2017; Liew *et al.*, 2012). HIF is a heterodimeric transcription factor consisting of an α (HIF-α) and a β (HIF-β) subunit. Both subunits are constitutively expressed; however, only the HIF-α is regulated, being rapidly degraded by an oxygen-dependent, proteasome-dependent mechanism (Kaluz *et al.*, 2006). HIF-α accumulation is correlated with cellular changes associated with aggressive tumour subtypes (Zhang *et al.*, 2005) and cancer treatment resistance (Masoud & Li, 2015). Hence, downregulation of HIF-α can be a target to reduce treatment resistance and improve therapeutic effects. Understanding the correlation between NDV infection and HIF-α expression will help improve treatment modalities and assist in the design of combination therapy in cancer.

The role of oncolytic viruses in the regulation of HIF is controversial. While some studies showed that oncolytic viruses more efficiently killed HIF-expressing cells (Connor *et al.*, 2004; Roos *et al.*, 2010), others showed the opposite (Hwang *et al.*, 2006). The varying outcomes are likely due to the pathotypes of the virus as well as the types of cancer cells used. NDV is categorized into three pathotypes, namely, lentogenic (non-virulent), mesogenic (intermediate) or velogenic (highly virulent) (Aldous & Alexander, 2001). Our recent report (Ch’ng *et al.*, 2013) using a local isolate of a viscerotropic–velogenic strain of NDV (Murulitharan *et al.*, 2013), designated as AF2240, showed that the virus displayed an increased oncolytic capacity in hypoxic cancer cells. To investigate the molecular mechanism of this observation, we performed NDV infection on selected cancer cell lines. In the present study, we used the osteosarcoma (Saos-2), breast carcinoma (MCF-7), colon carcinoma (HCT116) and fibrosarcoma (HT1080) cell lines.

## Results

### NDV infection diminished hypoxia-induced HIF-1*α* accumulation leading to decreased carbonic anhydrase IX expression

**Fig. 1.**
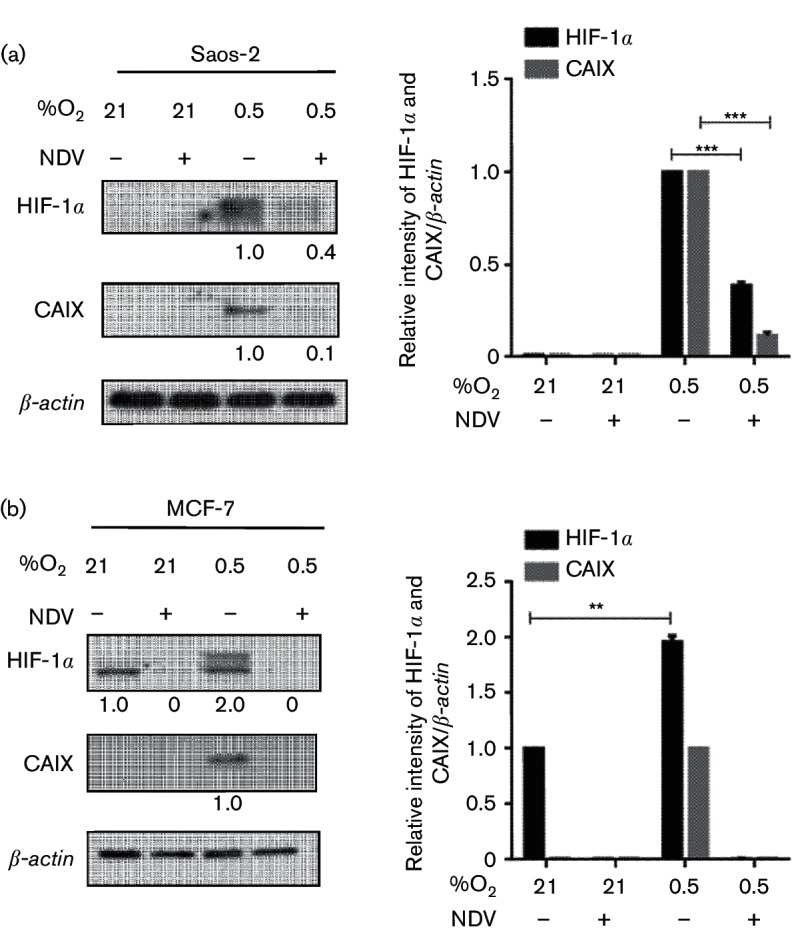
NDV infection diminished hypoxia-induced HIF-1α accumulation leading to decreased CAIX expression. Saos‑2 (a) and MCF‑7 (b) cells were cultured under normoxia (21 % O_2_) or hypoxia (0.5 % O_2_) in the presence or absence of NDV infection. Cells were harvested 25 h post-infection, and the levels of HIF‑1α and CAIX proteins were examined by immunoblotting. **P<0.01, ***P<0.001.

The correlation between oncolytic virus infection and HIF-α expression is controversial. Infection by a number of these viruses resulted in higher cytotoxicity in HIF-expressing cells (Connor *et al.*, 2004; Roos *et al.*, 2010), while others showed the opposite (Hwang *et al.*, 2006). The difference in the cytotoxicity level was associated with the level of HIF-α expression within the cells. Because of this conflicting evidence, we set out to investigate the effects of NDV infection on HIF-1α level and its regulation in cancer cell lines. Initially, we examined the effects of NDV infection on the levels of HIF-1α in normoxic and hypoxic cells. The absence of HIF-1α band in the normoxic Saos-2 cells remained the same following NDV infection (Fig. 1a). Interestingly, the level of hypoxia-induced accumulation of HIF-1α was significantly reduced in hypoxic Saos-2 cells following the infection. This reduction was correlated with a lowered level of carbonic anhydrase IX (CAIX) protein expression (Fig. 1a), of which the encoding gene, *carbonic anhydrase 9* (*CA9*), is a direct downstream target gene of HIF-1 (Abd-Aziz *et al.*, 2015; Grabmaier *et al.*, 2004; Raval *et al.*, 2005); hence, its expression is a demonstration of HIF-1α stabilization and, consequently, of HIF-1 transcriptional activity.

To examine whether this reduction of hypoxia-induced HIF-1α accumulation by NDV also occurs in other cancer cell lines, we repeated the experiment with MCF-7 breast adenocarcinoma cells. The MCF-7 cell line showed a high basal level expression of HIF-1α under normoxic condition (Fig. 1b); however, no CAIX was seen in the sample. It is interesting to note that, in this normoxic MCF-7 sample, only the lower molecular weight protein band of the HIF-1α was visible. Previous studies suggested that this band represented the native HIF-1α protein which lacked post-translational modification and was not transcriptionally active (Katschinski *et al.*, 2002). The basal level of HIF-1α in MCF-7 was significantly increased by hypoxia. In this sample, the higher molecular weight band of the HIF-1α was seen in addition to the lower band. This increase was correlated with the accumulation of CAIX. Following NDV infection, HIF-1α in MCF-7 under both conditions was diminished. This is evident from the disappearance of the HIF-1α band in cells infected with NDV. This absence of HIF-1α led to no detection of CAIX in the infected cells. This observation suggests that NDV was able to downregulate HIF-1α levels in both normoxic and hypoxic cells.

### NDV infection led to VHL degradation under normoxia and hypoxia without affecting viral protein synthesis and oncolytic activity

**Fig. 2.**
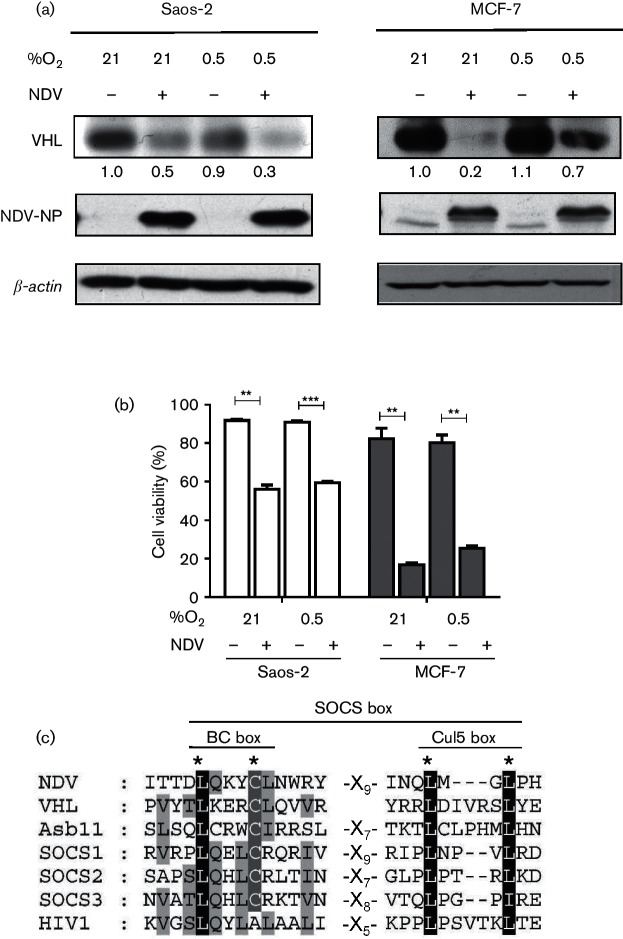
NDV infection led to a reduction of VHL protein but an increase in viral protein level. (a) Saos-2 and MCF-7 cells were infected with NDV AF2240 under normoxic (21 % O_2_) or hypoxic (0.5 % O_2_) conditions. Cell lysates were then probed for VHL and the NP viral protein. (b) Viability of cells in the cultures were evaluated by flow cytometry. (c) Alignment of the NDV L-proteins against selected SOCS box sequences revealed a possible conserved sequence. Dark shading indicates identical residues within the SOCS box. *P<0.05, **P<0.01.

Previously, we observed that NDV caused a slight but statistically significant higher level of oncolysis in hypoxic compared to normoxic renal carcinoma cell lines (Ch’ng *et al.*, 2013). In both conditions, renal cell carcinoma (RCC) cells devoid of VHL expression showed higher resistance to NDV killing. In VHL-reconstituted cells, NDV infection caused a significant reduction of VHL protein with only minimal influence on its oncolytic activity. To investigate whether the same event happens in the VHL wild-type MCF-7 and Saos-2 cells, harvested samples were probed for the VHL protein. Similar to Ch’ng *et al.* (2013), VHL protein levels in these two cells lines were also reduced following NDV infection in both normoxic and hypoxic conditions (Fig. 2a). The infection of the cells by NDV was confirmed by the detection of its nucleocapsid protein (NP). The level of this viral protein appeared to be at a similar level in both conditions. NDV-induced oncolytic activity was also seen to be similar (Fig. 2b). Since VHL degradation failed to restore HIF-1α accumulation as seen in Fig. 1, we investigated the possibility of a direct involvement of viral proteins in the degradation of VHL protein. Previously, in a RCC system, we reported a possible involvement of the suppressors of cytokine signalling (SOCS) proteins in NDV oncolytic activity (Ch’ng *et al.*, 2013). SOCS family of proteins contains the SOCS box motif, which allows them to function as substrate-recognition modules to mediate the cellular proteasomal process (Kamura *et al.*, 2004). In the present study, analysis of the sequences of the NDV proteins led us to discover a potential SOCS box motif in one of the viral proteins (Fig. 2c). This motif is positioned within the amino acids 638 to 669 of the large polymerase (L) protein (GenBank accession number AAP86958.1).

### NDV suppressed HIF-1*α* levels post-translationally, correlating with reduced *CA9* transcripts

**Fig. 3.**
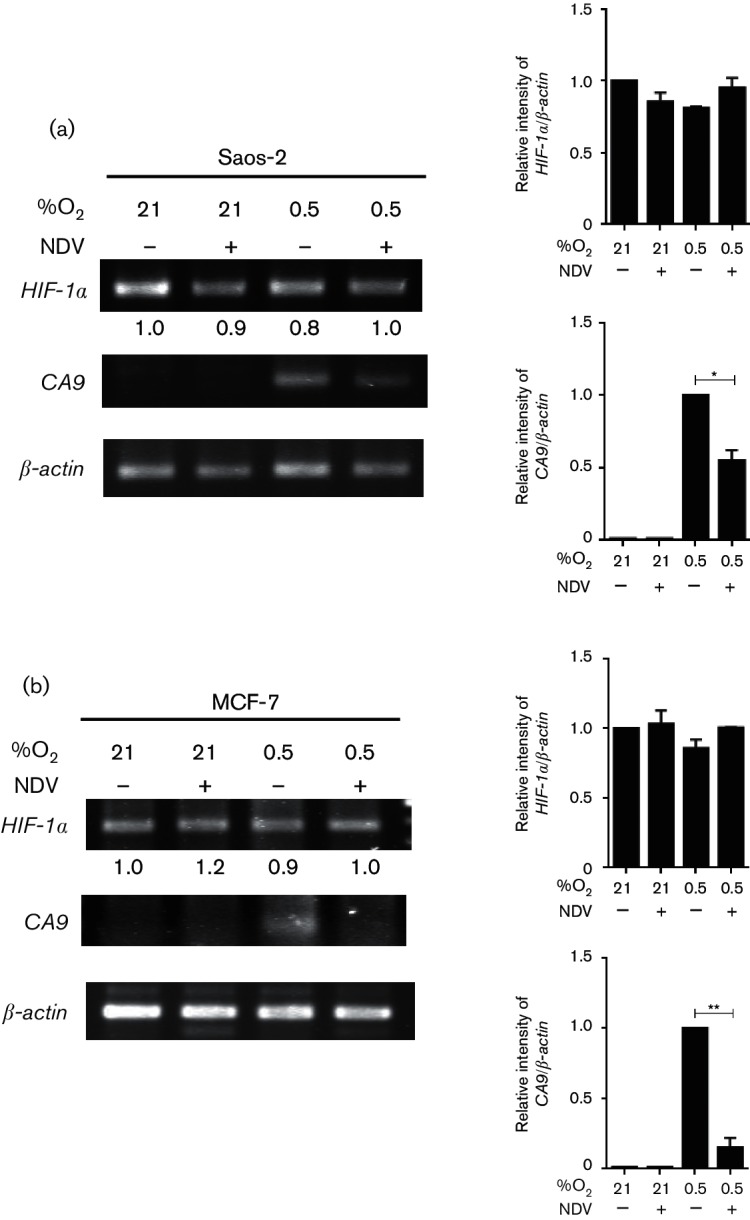
NDV suppressed HIF-1α level post-translationally, correlating with reduced *CA9* transcripts. Saos-2 (a) and MCF-7 (b) cells infected with NDV in either 21 % O_2_ or 0.5 % O_2_ condition. The levels of *HIF-1α* and *CA9* transcripts were examined from total RNA lysates of the samples. *P<0.05, **P<0.01.

To examine whether the suppression of HIF-1α in NDV-infected cells was regulated at its transcriptional or translational level, we performed reverse transcription PCR (RT-PCR) on total RNA samples from the infected and mock-infected Saos-2 and MCF-7 cells. A slight variation in the amplified *HIF-1α* band intensity was seen in the Saos-2 samples (Fig. 3a, left panel). However, when it was normalized to the *β-actin* loading control, no significant differences were seen between them (Fig. 3a, right panel).

To test whether the detected *HIF-1α* transcript was translated to a functional HIF-1α inside the cells, we performed RT-PCR using a set of primers for the *CA9* gene. *CA9* is a specific target of HIF-1 transcription factor complex (Grabmaier *et al.*, 2004; Kaluz *et al.*, 2006; Raval *et al.*, 2005). *CA9* transcript was not detectable in the normoxic samples regardless of whether they were infected with NDV (Fig. 3a). The band was only visible in the hypoxic samples. The intensity of this *CA9* band was significantly reduced in the hypoxic sample with NDV infection.

When the experiments were repeated in the MCF-7 cells, the patterns of *HIF-1α* and *CA9* bands were similar to the ones observed in the Saos-2 cells (Fig. 3b). The downregulation of *CA9* transcript level in both cell lines, when they were infected by NDV under hypoxia, was in line with the reduced CAIX protein expression levels observed in Fig. 1. These data suggest that the NDV-induced reduction of HIF-1α levels in Saos-2 and MCF-7 occurred post-translationally, resulting in a decrease in the transcription of their target gene, *CA9*.

### HIF-1*α* downregulation by NDV was partially abrogated by proteasomal inhibition in hypoxic condition

To investigate whether the reduced HIF-1α level in NDV-infected cells was due to a reduction of its translation level or an increase in its degradation, we used bortezomib. Bortezomib is the first proteasome inhibitor drug approved by the US Food and Drug Administration for anticancer treatment (Kane *et al.*, 2007). The rationale for using bortezomib is that, if HIF-1α protein translation is reduced in the presence of NDV infection, then an addition of bortezomib will not lead to its accumulation. Since HIF-1α degradation under normoxia is regulated through the proteasomal pathway (Masoud & Li, 2015), we were interested to see whether a similar pathway was also involved in the degradation of HIF-1α in NDV-infected cells. In this case, the addition of bortezomib would cause a restoration of HIF-1α level in the NDV-infected samples.

**Fig. 4.**
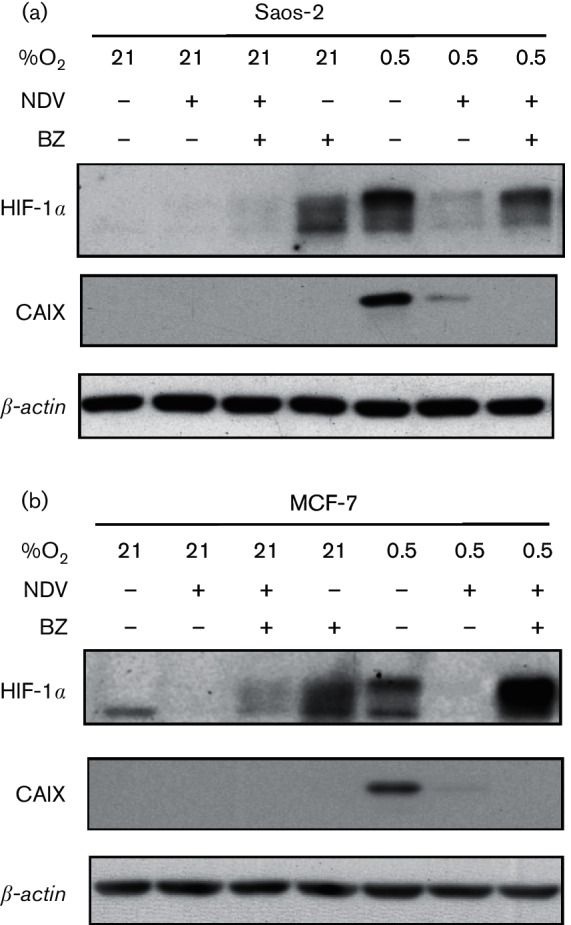
HIF-1α downregulation by NDV was partially abrogated by proteasomal inhibition in hypoxic condition. Saos-2 (a) and MCF-7 (b) cells were infected with NDV under 21 % O_2_ or 0.5 % O_2_ in the presence or absence of bortezomib (BZ). Samples were harvested at 25 h post-infection, and the levels of HIF-1α and CAIX proteins were examined.

In Saos-2, no HIF-1α was detected in the mock-infected normoxic samples (Fig. 4a). In MCF-7 cells, only the non-transcriptionally active lower molecular weight band of the HIF-1α (Katschinski *et al.*, 2002) was seen (Fig. 4b). In the presence of bortezomib, HIF-1α level was increased in both cell lines. This was expected since bortezomib inhibited the oxygen-regulated HIF-1α degradation via inhibition of the proteasomal pathway (Li *et al.*, 2008). NDV infection, however, caused this bortezomib-regulated normoxic stabilization of HIF-1α to disappear. This phenomenon was seen in both Saos-2 and MCF-7 cell lines. Hypoxia conditions caused HIF-1α to be stabilized in both cell lines. This stabilization was also abolished in the presence of NDV infection. Bortezomib addition resulted in a restoration of HIF-1α accumulation in NDV-infected hypoxic cells with a more drastic accumulation seen in MCF-7 samples.

To evaluate the functionality of HIF-1α towards HIF-1 transcriptional activity, the samples were probed for CAIX protein. HIF-1α accumulation caused by bortezomib addition did not lead to CAIX expression under either hypoxic or normoxic conditions. This observation agreed with a previous report of bortezomib attenuation of HIF-1 activity (Kaluz *et al.*, 2006). Only hypoxia-induced HIF-1α accumulation was associated with CAIX expression.

### NDV-induced HIF-1*α* downregulation is independent of p53

**Fig. 5.**
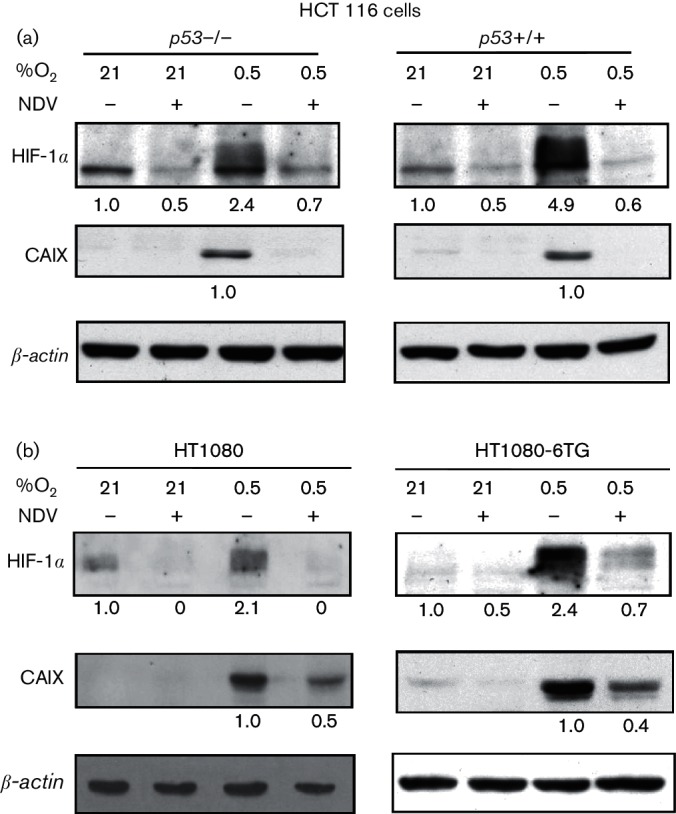
NDV-induced HIF-1α downregulation is independent of p53. We repeated the NDV infection experiments using *p53* null and *p53* wild-type HCT116 colon carcinoma cells (a) as well as the HT1080 (wild-type *p53* alleles) and HT1080.6TG (mutated *p53* alleles) cell lines (b). At 25 h post-infection, levels of HIF‑1α and CAIX proteins were examined with immunoblotting.

p53 is involved in HIF-1α regulation through direct association and parallel pattern protein stabilization (Piret *et al.*, 2002). Therefore, we wondered whether p53 is also involved in NDV-induced HIF-1α downregulation. We repeated the experiments using a *p53* null and a *p53* wild-type HCT116 colon carcinoma cell line (Vogelstein *et al.*, 2000). These cell lines displayed a basal level expression of the lower molecular weight non-transcriptionally active (Katschinski *et al.*, 2002) HIF-1α (Fig. 5a). This level is increased together with the appearance of the higher molecular weight HIF-1α when the cells were cultured in hypoxic condition. The presence of HIF-1α in both normoxic and hypoxic conditions was almost eliminated by NDV infection. This reduction caused the disappearance of CAIX expression in the cells.

Similar phenomena also occurred in the HT1080 and HT1080.6TG fibrosarcoma cell lines (Fig. 5b). The HT1080 cell line contains two wild-type p53 alleles (Tarunina & Jenkins, 1993), while the HT1080.6TG carries two mutated p53 alleles (Anderson *et al.*, 1994). In these cell lines, the hypoxia-induced HIF-1α stabilization was also abrogated when the cells were infected with NDV. These HIF-1α level changes correlated with their CAIX expression. Taken together, these data suggest that NDV-induced downregulation of HIF-1α is independent of wild-type p53 activity.

### Lentogenic NDV strain V4-UPM did not induce degradation of HIF-1*α* and VHL

**Fig. 6.**
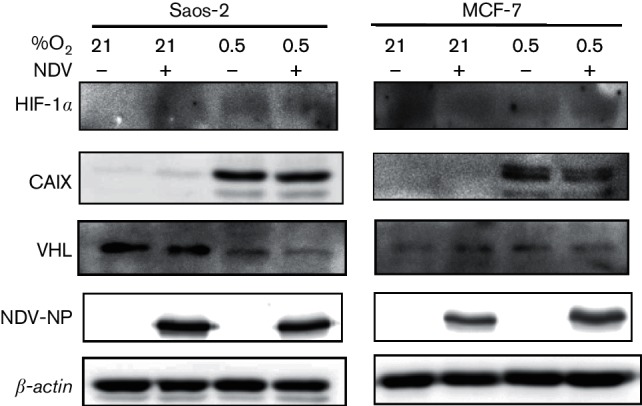
Lentogenic NDV strain V4-UPM did not induce degradation of HIF-1α and VHL. Cells were infected with a lentogenic strain of NDV, designated V4-UPM. Cell lysates were probed for specific antibodies towards HIF-1α, CAIX, VHL and the NP viral proteins.

Thus far, we showed that the AF2240 virulent strain of NDV caused degradation of HIF-1α and VHL in infected cancer cells. To examine whether a non-virulent strain of NDV also has the same capability, we performed infections using the V4-UPM strain of NDV (Alabsi *et al.*, 2012). Results obtained showed that this NDV strain was not able to cause degradation of HIF-1α and VHL in either Saos-2 or MCF-7 cell line (Fig. 6). The lack of HIF-1α degradation was also evident by the similar level of CAIX protein in the infected and mock-infected hypoxic samples.

## Discussion

Viral infection and replication are significantly influenced by oxygen tension (Morinet *et al.*, 2013). The cytotoxic effects of a number of oncolytic viruses are affected by hypoxia and the level of HIF in the infected cells (Cho *et al.*, 2010; Connor *et al.*, 2004; Hiley *et al.*, 2010). Viral infection itself caused deregulation of HIF levels within hypoxic cancer cells (Gupta-Saraf & Miller, 2014), resulting in the varying regulation of their downstream target genes (Cho *et al.*, 2010). In the present study, we showed that a velogenic NDV infection led to a downregulation of HIF-1α protein in a p53-independent manner. NDV-induced downregulation of HIF-1α seen in the present study agrees with a number of previous reports on viral infection data. Moloney murine leukaemia temperature-sensitive virus (Lungu *et al.*, 2008) and reovirus (Cho *et al.*, 2010) infections were shown to cause downregulation of HIF-1α expression. On the contrary, other viruses, such as Kaposi’s sarcoma-associated herpesvirus and human herpesvirus-8, caused HIF-1α to be accumulated in infected cells in normoxic conditions (Cai *et al.*, 2007; Carroll *et al.*, 2006).

Besides HIF-1α degradation, surprisingly, we also observed that the VHL protein was likewise degraded. This observation suggests that the NDV-induced HIF-1α degradation was independent of VHL. Despite the reduction of HIF-1α and VHL protein levels, viral protein production was increased in the infected cells. The appearance of a reverse correlation between HIF-1α and NDV proteins suggests a possible link between them. In addition, the observation of VHL degradation in the absence of HIF-1α stabilization suggests a more direct role of NDV proteins in the HIF-1α degradation process. In the present study, analysis of the NDV protein sequences led us to discover a potential SOCS box motif in its L-protein (amino acids 638 to 669). This novel observation highlights the potential of this L-protein of NDV to be a substrate-recognition protein in the process of proteasomal degradation. This possibility is reinforced by a report that showed the expression of SOCS-domain-containing viral proteins led to VHL degradation (Pozzebon *et al.*, 2013). In agreement with this, our previous study of NDV infectivity in RCC cell lines (Ch’ng *et al.*, 2013) also revealed the involvement of the SOCS family of proteins. The presence of a SOCS domain in the L-protein of NDV is not surprising, since besides its known function as an RNA polymerase, it was also reported to be involved in other enzymatic activities to assist NDV gene transcription and viral replication (Poch *et al.*, 1990). We are currently cloning the L gene and its truncations and will subject them to various mutational analyses. For now, however, we can only speculate on the possible function of the L-protein in host protein degradation, in particular the HIF-1α and VHL proteins.

In the present study, we showed that a velogenic NDV infection caused a decrease in HIF-1α protein level, but did not affect its level of transcription. These results suggest that NDV-induced downregulation of HIF-1α occurs post-translationally. In a reovirus infection study, Cho *et al.* (2010) reported that the virus caused HIF-1α reduction also at a post-transcriptional level. Although they showed a reduction of *VEGF* and *Glut-1*, which are HIF-responsive genes that can also be activated by other transcription factors (Cho *et al.*, 2010), they did not examine the level of *CA9*, a HIF-1-specific target gene (Abd-Aziz *et al.*, 2015; Grabmaier *et al.*, 2004; Raval *et al.*, 2005). In our study, we show that the downregulation of HIF-1α protein by NDV led to a reduced *CA9* transcript level, causing a reduction in CAIX expression. This novel observation highlights the potential use of NDV as a specific target for CAIX suppression in addition to its oncolytic effects. CAIX has been a major target in cancer therapeutics (McDonald *et al.*, 2012). This is due to the fact that CAIX overexpression is predominantly associated with poor prognosis, particularly in hypoxic tumours (Driessen *et al.*, 2006; Huang *et al.*, 2015). CAIX, a carbonic anhydrase, is an important regulator of cellular pH during hypoxia (Dubois *et al.*, 2011; Swietach *et al.*, 2007). Reduction or inhibition of CAIX resulted in decreased cell proliferation while increasing apoptosis (Cianchi *et al.*, 2010; Dubois *et al.*, 2011). It also has been shown to augment the effects of radiation and chemotherapy (Proescholdt *et al.*, 2012).

Cellular protein concentrations are controlled by a balance between their synthesis and degradation. Unwanted proteins are degraded through the proteasome proteolytic pathway. This pathway is an important part of the viral replication process within infected cells (Luo *et al.*, 2003; Zhang *et al.*, 2004). Viruses commandeer proteasomal functions to regulate cellular machinery and favour viral replication. To investigate whether this pathway is involved in NDV-induced HIF-1α degradation, we utilized a proteasome inhibitor drug, bortezomib (Kane *et al.*, 2003, 2007). Our RT-PCR data suggested that *HIF-1α* was continuously transcribed in the presence of NDV infection. In uninfected cells, the transcript is subsequently translated and subjected to post-translational modifications, resulting in an active stable HIF-1α subunit. This process was interrupted by NDV infection where HIF-1α stabilization in hypoxic cells was diminished. Bortezomib treatment of NDV-infected hypoxic cells abrogated the NDV-induced HIF-1α downregulation. Overall, this observation suggested that the NDV-induced downregulation of HIF-1α occurred through the proteasomal pathway. Since bortezomib caused accumulation of transcriptionally inactive HIF-1α (Kaluz *et al.*, 2006), no downstream target gene, in this case *CA9* (Simon *et al.*, 2005), was upregulated. p53 is involved in HIF-1α regulation under various physiological conditions (Schmid *et al.*, 2004). It is also involved in cellular responses to viral infections (Luke, 2008). In the present investigation, we noted that cell lines that are *p53* mutated and *p53* null also resulted in HIF-1α downregulation upon NDV infection. This evidence suggested that NDV-induced HIF-1α downregulation is independent of p53 activity. This information may be useful in designing combination therapy involving oncolytic viruses in cancers, irrespective of p53 status.

## Methods

### Cell lines, NDV and culture conditions.

Osteosarcoma (Saos-2), breast carcinoma (MCF-7), colon carcinoma (HCT116) and fibrosarcoma (HT1080) cell lines were maintained in Dulbecco’s modified Eagle’s medium supplemented with 10 % FBS (PAA) at 37 °C in a humidified CO_2_ incubator. Prior to cell seeding for NDV infection studies, the cells were subcultured three times at 1×10^4^ cm^−2^ (Abd-Aziz *et al.*, 2015). Cells were then initially seeded at 2×10^4^ cm^−2^ and incubated overnight. They were then infected with either a velogenic NDV strain, designated as AF2240 (Yusoff & Tan, 2001), or a lentogenic strain, V4-UPM (Alabsi *et al.*, 2012), as described previously (Ch’ng *et al.*, 2015; Chia *et al.*, 2012). In cases where cell viability measurement was needed, flow cytometry was used (Ch’ng *et al.*, 2013). Time zero or 0 h post-infection began after a 1 h virus adsorption period. In bortezomib-treated samples, the drug was mixed (at the required final concentration) with the culture medium that was added to the cells following the 1 h adsorption period. The infected cells were subsequently placed in a humidified CO_2_ incubator for normoxia samples (21 % O_2_) or a Galaxy 48R incubator (New Brunswick) for hypoxia (0.5 % O_2_) samples.

### Protein harvesting, separation and immunoblotting.

Cells were harvested on ice using radioimmunoprecipitation assay buffer (Thermo Scientific), containing EDTA-free protease inhibitor cocktail (Roche), and separated by SDS-PAGE. After they were electro-transferred onto a PVDF membrane (Pall Corporation), the samples were probed using antibodies against HIF-1α, HIF-2α, CAIX (all from Genetex), VHL (BD Pharmingen), anti-NDV (obtained from Universiti Putra Malaysia) and β-actin (Sigma-Aldrich) as described by the manufacturers. The primary antibodies were then detected with HRP-conjugated secondary antibodies (Cell Signaling Technology). Protein bands were visualized using the SuperSignal West Dura Extended Duration Substrate kit (Pierce Biotechnology) and quantitated as described previously (Ch’ng *et al.*, 2013).

### RT-PCR.

Total RNA samples were isolated from the infected and control cells using the total RNA purification kit (Norgen). RT-PCRs were performed on 100 ng RNA using the Access RT-PCR system (Promega). Specific sets of primers for *HIF‑1α*, *CA9* and *β-actin* (Kaluz *et al.*, 2006) were used. The reaction mixture contained 1× AMV/Tfl reaction buffer, 10 mM dNTP mix, Tfl DNA polymerase (0.1 U), AMV RT (0.1 U), 25 mM MgSO_4_ and 10 mM forward and reverse primers. The reaction cycle included 1 cycle of reverse transcription at 45 °C for 45 min, 1 cycle of pre-denaturation at 94 °C for 2 min, followed by 30 cycles (with the exception of β-actin, 25 cycles) at 95 °C for 40 s, 56 °C for 40 s and 72 °C for 1 min. A final step at 72 °C for 4 min concluded the reaction cycle. The resulting gene products were analysed on 1.5 % agarose gel and quantitated using the ImageJ 1.48 software (Wayne Rasband, NIH).

### Statistical analysis.

Experimental data were analysed using the Student’s *t*-test (GraphPad Prism 5; GraphPad Software) and expressed as mean±sem. Statistical significance was defined as *P*<0.05.
